# Microglia orchestrate neuroinflammation

**DOI:** 10.7554/eLife.81890

**Published:** 2022-08-22

**Authors:** Ricardo A Feldman

**Affiliations:** 1 Department of Microbiology and Immunology, University of Maryland School of Medicine Baltimore United States

**Keywords:** Gaucher disease, neuroinflammation, microglia, Gba1, NK cells, lipids, Human, Mouse

## Abstract

Experiments in genetically altered mice reveal that microglia play an important role in the neurological damage associated with neuro-nopathic Gaucher disease.

**Related research article** Boddupalli CS, Nair S, Belinsky G, Gans J, Teeple E, Nguyen TH, Mehta S, Guo L, Kramer ML, Ruan J, Wang H, Davison M, Kumar D, Vidyadhara DJ, Zhang B, Klinger K, Mistry PK. 2022. Neuroinflammation in neuronopathic Gaucher disease: Role of microglia and NK cells, biomarkers, and response to substrate reduction therapy. *eLife* **11**:e79830. doi: 10.7554/eLife.79830.

Gaucher disease is a genetic disorder caused by mutations in the gene coding for the enzyme glucocerebrosidase. These mutations prevent cells from breaking down a lipid called glucosylceramide, which, together with its metabolite glucosylphingosine, promotes inflammation and other alterations that can harm the body’s tissues (Grabowski et al., 2021; Nagata et al., 2017; Nair et al., 2015).

There are three different forms of Gaucher disease: type 1 which is milder and affects the spleen, liver and bone; and types 2 and 3 which, in addition to these alterations, damage neurons and other cell types in the brain. However, the mechanisms responsible for the neurological effects associated with types 2 and 3 (which are collectively known as neuronopathic Gaucher disease) are poorly understood. This is largely because neurodegeneration is difficult to study as it involves complex interactions between neurons and multiple other cell types in the brain, including microglia and astrocytes, which support the function of neurons. Now, in eLife, Pramod Mistry and colleagues from Yale University – including Chandra Sekhar Boddupalli and Shiny Nair as joint first authors – report that microglia play a central role in the neurological damage associated with neuronopathic Gaucher disease (Boddupalli et al., 2022).

The team set out to find how glucocerebrosidase deficiency leads to neuroinflammation which is a major determinant of neurodegeneration in Gaucher disease. To do this, Boddupalli et al. studied a mouse model of neuronopathic Gaucher disease which lacked the gene for glucocerebrosidase (called *Gba1*) in all tissues except skin to avoid death soon after birth. They also generated three additional mouse models: neuronopathic Gaucher disease mice where *Gba1* expression was restored in their microglia or neurons, and mice with *Gba1* selectively deleted from their microglia, which mimics late onset neuronopathic Gaucher disease.

Boddupalli et al. found that loss of *Gba1* resulted in microglia and astrocyte activation, as well as blood-derived immune cells infiltrating the brain (Figure 1A). Analyzing which genes were expressed in the different cell types of *Gba1* deficient mice at a single cell resolution revealed that important neuroinflammatory networks became uncontrolled. In particular, the activated microglia expressed a set of genes that triggered an immune response. However, when the expression of *Gba1* was restored in microglia, this reduced inflammation and astrocyte immune activation, stemmed the influx of immune cells, and improved mouse survival (Figure 1B). These results suggest that while the infiltration of immune cells into the brain is a key aspect of neuronopathic Gaucher disease, microglia activation plays a prominent role in the neuroinflammation associated with the disorder.

**Figure 1.**
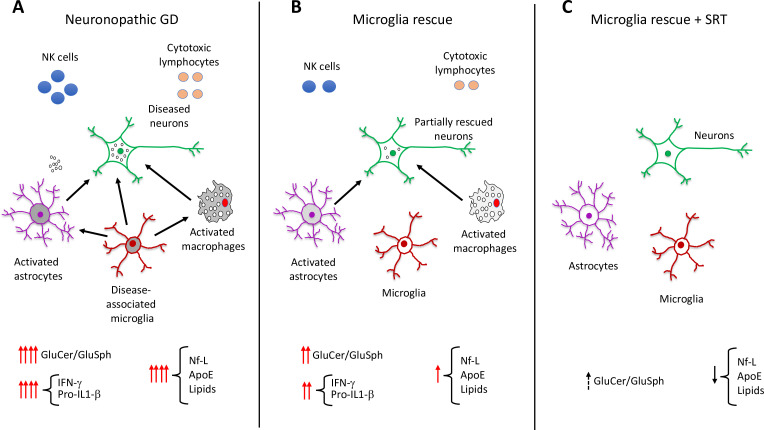
Role of elevated glucosphingolipids and activated microglia in neuronopathic Gaucher disease. (**A**) In the brains of neuronopathic Gaucher disease mice are multiple interacting cell types, including neurons (green outline), astrocytes (purple outline), microglia (red outline), and immune cells from the blood, such as macrophages (grey), NK cells (blue circles), and cytotoxic lymphocytes (orange circles). Neuronopathic Gaucher disease mice, which lack the *Gba1* gene, have higher levels of glucosylceramide and glucosylsphingosine. Elevation of these glucosphingolipids deregulates the interactions (black arrows) between neurons, microglia, astrocytes and macrophages, causing the immune cells to release inflammatory cytokines such as IFN-γ and Pro-IL-1β. The resulting inflammatory environment leads to neuronal injury and death. Injured neurons release the biomarker Nf-L, and there are also higher levels of the lipoprotein ApoE and certain lipids (such as hexosylceramides and lysophosphatidylcholine), which are released into the blood where they can be detected. (**B**) Selective rescue of *Gba1* expression in the microglia of neuronopathic Gaucher disease mice reduced the influx of blood-derived immune cells, and the immune activity of macrophages and astrocytes. This decreased the levels of inflammatory cytokines, resulting in less neuronal injury and lower levels of the biomarkers Nf-L, ApoE, and lipids. (**C**) Substrate reduction therapy (SRT) using a drug that blocks the synthesis of glucosylceramide is approved to treat patients with type 1 Gaucher disease. When mice with *Gba1* expression restored in their microglia were treated with a brain-permeable SRT drug, the level of glucosphingolipids and neuroinflammation was reduced even further, along with a decrease in the levels of Nf-L, ApoE and lipid biomarkers. GluCer, glucosylceramide; GluSph, glucosylsphingosine; Nf-L, neurofilament light chain; ApoE, apolipoprotein E; IFN-γ,Interferon gamma; Pro-IL-1-β, Pro-Interleukin 1 beta.

Image credit: Ricardo A. Feldman.

Next, Boddupalli et al. investigated the effects of substrate reduction therapy (SRT), a treatment strategy for certain metabolic disorders. SRT drugs block the synthesis of glucosylceramide, preventing this lipid and its metabolite glucosylsphingosine from accumulating inside cells (Blumenreich et al., 2021; Cabrera-Salazar et al., 2012). An SRT approach using a drug that cannot cross the blood-brain-barrier has been approved to treat the non-neurological symptoms of Gaucher disease. Boddupalli et al. found that administration of a brain-penetrant SRT drug to mutant mice that re-expressed *Gba1* in their microglia, reduced neuroinflammation even further (Figure 1C). These findings suggest that the accumulation of glucosylceramide and glucosylsphingosine, and microglia activation, are the main drivers of neuroinflammation in Gaucher disease. A treatment that counteracts both these mechanisms could potentially ameliorate the neurological effects of the disorder.

Remarkably, the team also found a number of early indicators of neurological damage in mice lacking *Gba1*, including a marker of neuronal injury called Nf-L (short for neurofilament light chain; Gaetani et al., 2019; Weinhofer et al., 2021). Nf-L was 2,000 times higher in the blood of neuronopathic Gaucher mice, 100 times higher in mice with *Gba1* selectively deleted from their microglia, and this correlated with the elevation of glucosylphingosine, a well-established marker of Gaucher disease. In addition, patients with Gaucher disease type 3 had greater amounts of Nf-L in their blood than patients with type 1, who do not exhibit overt neurological symptoms.

A protein that plays a critical role in lipid metabolism called ApoE (short for apolipoprotein E; Serrano-Pozo et al., 2021) was also highly elevated in the astrocytes and microglia of the mutant mice, as well as in the blood of untreated type 1 Gaucher patients. Furthermore, other lipids, namely hexosylceramides and lysophosphatidylcholine (Bodennec et al., 2002), were present at very high levels in the brains of mice which had *Gba1* selectively deleted from their microglia. Boddupalli et al. found that SRT lowered the levels of Nf-L, ApoE, hexosylceramides and lysophosphatidylcholine in the mutant mice, providing further evidence that these molecules are relevant markers for neuronopathic Gaucher disease.

The lack of early biomarkers for neuronopathic Gaucher disease severely curtails clinicians’ ability to detect neurological damage before the onset of symptoms. Further validation of the markers identified in this study could help develop new therapies for preventing or delaying disease progression caused by glucocerebrosidase deficiency.

The potential impact of these findings extends well beyond Gaucher disease, as patients with this disorder have a five- to twenty-fold increased risk of developing Parkinson’s disease and Lewy Body Dementia. Furthermore, carriers of Gaucher disease, who have single allele mutations in the gene for glucocerebrosidase and no symptoms, are also equally susceptible to these neurodegenerative diseases (Aharon-Peretz et al., 2004; Sidransky and Lopez, 2012). Thus, early detection of the biomarkers identified could help clinicians detect which individuals are more likely to develop Parkinson’s disease and Lewy Body Dementia, and prevent or limit progression of these disorders.
